# Economic Potential for Distributed Manufacturing of Adaptive Aids for Arthritis Patients in the U.S.

**DOI:** 10.3390/geriatrics3040089

**Published:** 2018-12-06

**Authors:** Nicole Gallup, Jennifer K. Bow, Joshua M. Pearce

**Affiliations:** 1 Department of Biomedical Engineering and Mechanical Engineering, Michigan Technological University, Houghton, MI 49931, USA; ngallup@mtu.edu; 2 Department of Materials Science & Engineering, Michigan Technological University, Houghton, MI 49931, USA; jbow@mtu.edu; 3 Department of Electrical & Computer Engineering, Michigan Technological University, Houghton, MI 49931, USA; 4 Department of Electronics and Nanoengineering, School of Electrical Engineering, Aalto University, Espoo FI-00076, Finland

**Keywords:** 3-D printing, additive manufacturing, arthritis, adaptive aid, distributed manufacturing, economics, motor skills, person–environment interaction, cost-effective

## Abstract

By 2040, more than a quarter of the U.S. population will have diagnosed arthritic conditions. Adults with arthritis and other rheumatic conditions earn less than average yet have medical care expenditures that are over 12% of average household income. Adaptive aids can help arthritis patients continue to maintain independence and quality of life; however, their high costs limit accessibility for older people and the poor. One method used for consumer price reduction is distributed manufacturing with 3-D printers. In order to assess if such a method would be financially beneficial, this study evaluates the techno-economic viability of distributed manufacturing of adaptive aids for arthritis patients. Twenty freely accessible designs for 3-D printable adaptive aids were successfully fabricated on low-cost desktop 3-D printers and performed their functions adequately. The financial savings averaged >94% compared to commercially-available products. Overall, twenty adaptive aids were printed for US$20 of plastic; while on average, each adaptive aid would save over US$20. As printing a tiny subset of the adaptive aids needed by a single patient would recover the full capital and operational costs of a low-cost 3-D printer, it can be concluded that there is considerable potential for distributed manufacturing to assist arthritis patients.

## 1. Introduction

Arthritis is a common and disabling chronic condition consisting of joint pain and physical disability that has negative effects on the quality of life of patients [1,2]. In the U.S., this public health challenge is growing worse. On average, 54.4 million American adults had doctor-diagnosed arthritis in 2015, which accounts for 22.7% of the adult population [3]. Of those, 23.7 million (43.5% of those with arthritis) had arthritis-attributable activity limitations, which is about a 20% age-adjusted increase in the proportion of adults with arthritis reporting activity limitations since 2002 [3]. The Center for Disease Control estimates that by 2040, more than a quarter (25.9%) of the U.S. adult population (78 million) aged 18 years or older will have doctor-diagnosed arthritis [4,5].

Arthritis has a substantial economic cost for Americans. Adults with arthritis and other rheumatic conditions incurred mean medical care expenditures of $6,978 in 2003 [6], which is over 12% of the average household income for the same year [7]. Overall, expenditure for adults with arthritis was $321.8 billion in 2003 [6]. These expenditures, however, do not capture the complete economic picture for those with arthritis. First, those 18–64 years old with arthritis and other rheumatic conditions earned $3,613 less than expected, and $1,590 was attributable to arthritis and other rheumatic conditions [6]. Second, those with arthritis often have additional expenses that do not show up on medical bills and are not covered by insurance. For example, those who suffer from arthritis of the hands are challenged by even simple activities of daily living tasks (ADLs), such as opening jars, as this can be cumbersome and painful. There are adaptive aids available on the market to help with these tasks, but they are far more costly than conventional products. For example, a simple adaptive aid commercial spoon costs US$24.95 [8], which is more than 43 times more expensive than standard spoons available on the largest online retailer in the U.S. [9]. For those with arthritis, the additional financial strain to purchase all of the adaptive aids necessary to prevent limitations to independence can be substantial. In addition, prices for many consumer goods including adaptive aids are set to increase because of an ongoing trade war with China [10] and America’s withdrawal from a United Nations treaty that offered low rates for foreign postal deliveries of small packages in the U.S. [11] (e.g., adaptive aids purchased on E-Bay and shipped from overseas). Arthritis patients would benefit financially from price relief for adaptive aids which, even if the patient has health insurance, may not be covered by their health insurance or may have to be paid out of pocket under the patient’s health insurance deductible. Arthritis prevalence increases with age and the elderly population is already financially burdened. Under the supplemental poverty measure (SPM), 7.1 million adults ages 65 and older lived in poverty in 2016 (14.5%) [12]). Thus, there is a clear need to reduce the costs of adaptive aids for arthritis patients.

One method gaining favor for price reduction in consumer goods is distributed manufacturing of the goods with digital technologies such as 3-D printers [13,14,15,16]. 3-D printers can be used for distributed additive manufacturing either by local businesses [17,18,19,20], chain stores (e.g., shipping firms [21] or home improvement retailers [22]), or fab labs [23], makerspaces [24,25] and libraries [26]. Recent applications of free and open-source hardware (FOSH) development [27,28,29] have decreased the costs of 3-D printers [30] by providing self-replicating rapid prototypers (RepRaps) (e.g., 3-D printers that can largely print their own components) [31,32,33]. Several studies have shown that 3-D printers are a profitable investment for household-level distributed manufacturing [34,35,36,37]. Recently, Makers Making Change (www.makersmakingchange.com), an organization that connects people with disabilities that need assistive technologies (such as arthritis patients) with makers, was developed to design and fabricate assistive technologies. One way such an organization can scale laterally, and have their work help many that suffer from the same problem, is to distribute their technologies with open licenses so they can be utilized in distributed manufacturing [38,39].

In order to assess if such a method would be financially-viable for those with arthritis, this study evaluates the techno-economic viability of distributed manufacturing of adaptive aids for arthritis patients and specifically focuses on arthritis of the hands because the aids would be most likely to be able to be manufactured on a desktop 3-D printer. Twenty 3-D printable adaptive aids, which are provided to the global commons under free and open-source licenses, were fabricated on low-cost desktop 3-D printers in order to determine the distributed manufacturing costs. These products were tested for function and compared to those that offer similar functions on the U.S. market. These results are evaluated to determine the potential for distributed manufacturing to assist arthritis patients and conclusions are drawn.

## 2. Materials and Methods

### 2.1. Materials

For this analysis to be widely applicable, it was critical that the materials were relevant and accessible to the average consumer with arthritis or those that would be helping them, i.e., Makers Making Change. The most common desktop 3-D printing polymer is polylactic acid (PLA) and was selected for this study. PLA is popular because the deformation of the material is well understood [40] and it demonstrates less warping during printing than other materials such as the second most common 3-D printing plastic, acrylonitrile butadiene styrene or ABS. In addition, the emissions during 3-D printing for PLA are less pungent than ABS [41,42]. Lastly, PLA is made from corn-based resin, which is non-toxic, biodegradable, and able to be produced in a relatively environmentally-friendly and renewable processes [43,44] in addition to being able to be recycled back into 3-D printing filament [45,46,47]. 1.75 mm diameter PLA filament is available from most 3-D printing suppliers in the U.S. in prices ranging from US$10–50/kg.

### 2.2. 3-D Printers

The 3-D printers used for this study were delta-style RepRaps [48,49], which can be purchased in the Athena II kit form for under US$500 (3D4EDU, Houghton, MI, USA). The printers had a print volume: 220 mm diameter by 210 mm high semi-cylindrical shape, which was adequate to print all of the selected adaptive aids. The systems are completely open source, licensed under the GNU Affero General Public License as published by the Free Software Foundation [50]. The design files (STLs) were sliced with the open-source slicer Cura [51] using the settings recommended by the designer for a given adaptive aid. The 3-D printers were used in ambient indoor conditions with no heated bed nor enclosure to reduce the costs and guarantee realistic operating conditions and mechanical properties (e.g., tensile strength ranging from about 35 MPa to over 60 MPa) [52]. These mechanical properties of PLA would be expected to enable most load bearing applications in the class of adaptive aid for hand arthritis with appropriate fill settings. The 3-D printers with the web-based open-source Franklin CNC control software [53]. No post processing other than manual support material removal and assembly were conducted.

### 2.3. Adaptive Aid Selection

Twenty adaptive aids designed to help those with arthritis to accomplish a wide-array of routine ADLs were selected for inclusion in the study using the following six criteria:

1. They had freely available STL posted on an open-source 3-D printing design website MyMiniFactory (www.myminifactory.com). MyMiniFactory was used because all of the designs have already been pre-screened to be 3-D printable by MyMiniFactory staff.
2. They contained the source code (e.g., CAD design file from an open source and thus accessible CAD software package). This allows for relatively straight-forward customization of the device for specific people needing the aid.
3. They were licensed with an open-source compatible license or designer requests as defined by the Open Source Hardware Association [54].
4. They were able to be printed on a consumer grade 3-D printer (e.g., no high-cost precision metal 3-D printing or large scale printing).
5. They were able to be printed and used if manufactured from PLA (e.g., no specialty materials or applications that involved severe chemical compatibility).
6. They possessed a functionally equivalent commercialized proprietary product or a clear application for arthritis patients.

The selected open-source 3-D printable adaptive aids for arthritis patients are shown in Table 1 along with a brief description the type of open-source computer aided design (CAD) software used to design them and their sources.

### 2.4. Arthritis Patient Economics

For the adaptive aids selected in Table 1, functionally-equivalent commercialized products were found with Internet market research. A spreadsheet (Table S1) of all links to where the commercial equivalents can be purchased and where retail prices were found is in Supplementary Materials. The shipping costs associated with the products were excluded from the analysis for both purchasing commercial as well as distributed manufacturing (e.g., no shipping charges included for plastic filament) as free shipping is common with many online vendors in the U.S.

The economic inputs for distributed manufacturing are polymer feedstock, electricity and labor. The price of 1 kg of 1.75 mm diameter PLA filament is US$19.99 [55] (or the cost per gram, c = 2 cents/g). A digital scale with an error of ±0.05 g was used to mass each item after it was 3-D printed. The average residential electricity rate for the United States in August 2018 was US$0.133/kWh [56]. The energy use of the 3-D printer was measured with a multi-meter with a reading accuracy of 0.5%.

With the assumptions that labor costs and electricity costs were excluded as detailed below, the operating cost (O) for the 3-D printer for a given adaptive aid was calculated as:

(1) $$O_{a}=c\times m_{a}\;[\mathrm{US}\$]$$

where *m* is the 3-D printed mass of the parts for the adaptive aid in grams and *c* the cost per gram is given above. As *m* is the primary determinant of the economics of the adaptive aids an experiment was run on 150 replicates of an ASTM Type IV tensile bar from thirty new (<2 months of experience) delta RepRap users each printing five samples with a random selection of slicing settings with 100% fill. The new users were chosen to simulate new arthritis patients with a small or no 3-D printing experience. The slicing settings changed print speed (nozzle speed over the print bed), layer height, and nozzle temperature in such a way as to obtain a visually acceptable 3-D print. These changes could be expected to be made by users even if they used recommended print settings that are more important for the mechanical integrity of the device such as fill percentage and shell thickness. This experiment was used to determine the potential error in the operating cost from Equation (1).

The total cost for a given adaptive aid manufactured using a distributed model (*T_a_*) includes the cost of purchased components (*p*) (e.g., nuts and bolts):

(2) $$T_{a}=p_{a}+\;O_{a}\;[\mathrm{US}\$]$$

The marginal savings on each adaptive aid, $S_{a}$, is given by:

(3) $$S_{a}=C-T_{a}\;\;[\mathrm{US}\$]$$

where C is the cost of the commercially available product. Finally, the marginal percent change, φ, between the cost to 3-D print an adaptive aid and the commercially available product was calculated as follows:

(4) $$\varphi =\;\left(\frac{C-T_{a}}{C}\right)\;\times 100\;[\%]$$

## 3. Results

### 3-D Printable Adaptive Aids

**Figure 1:**
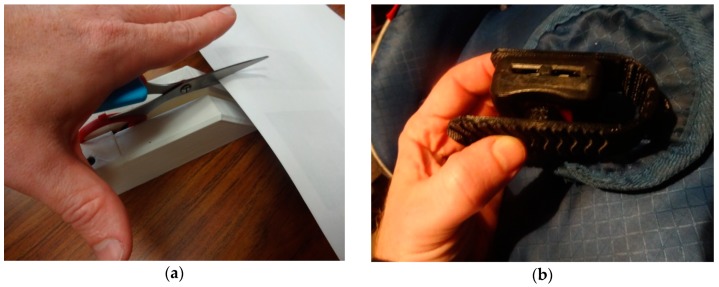

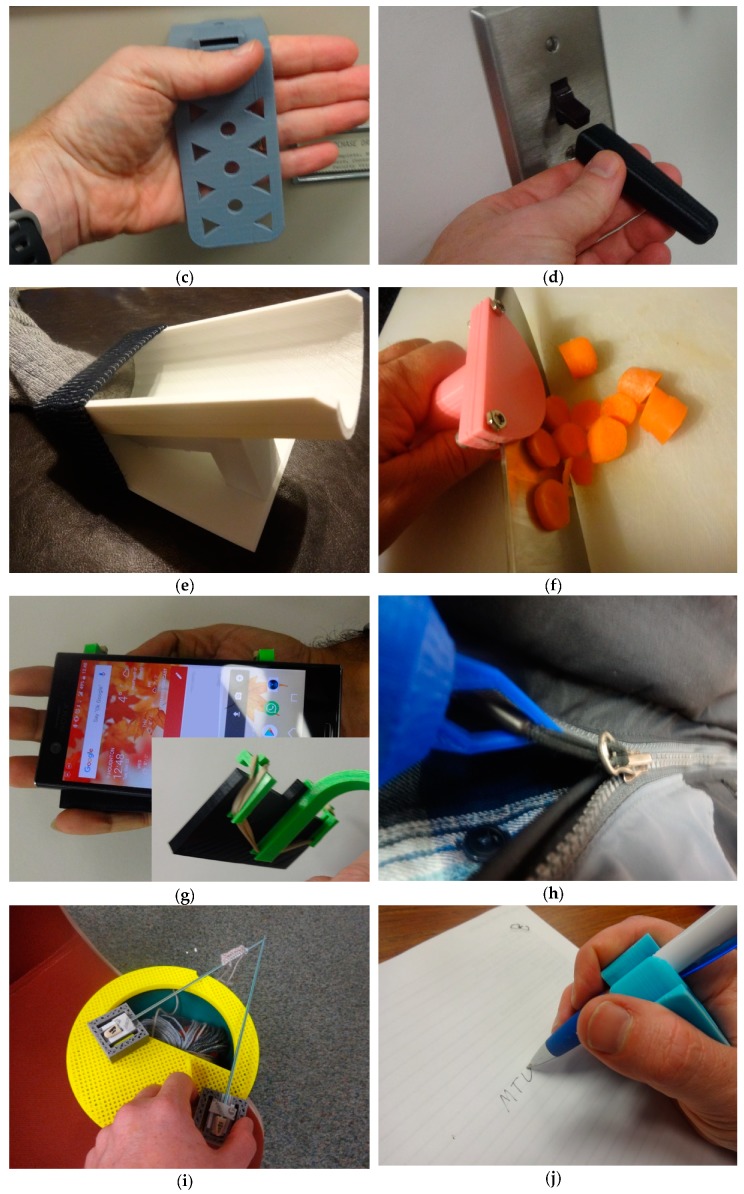

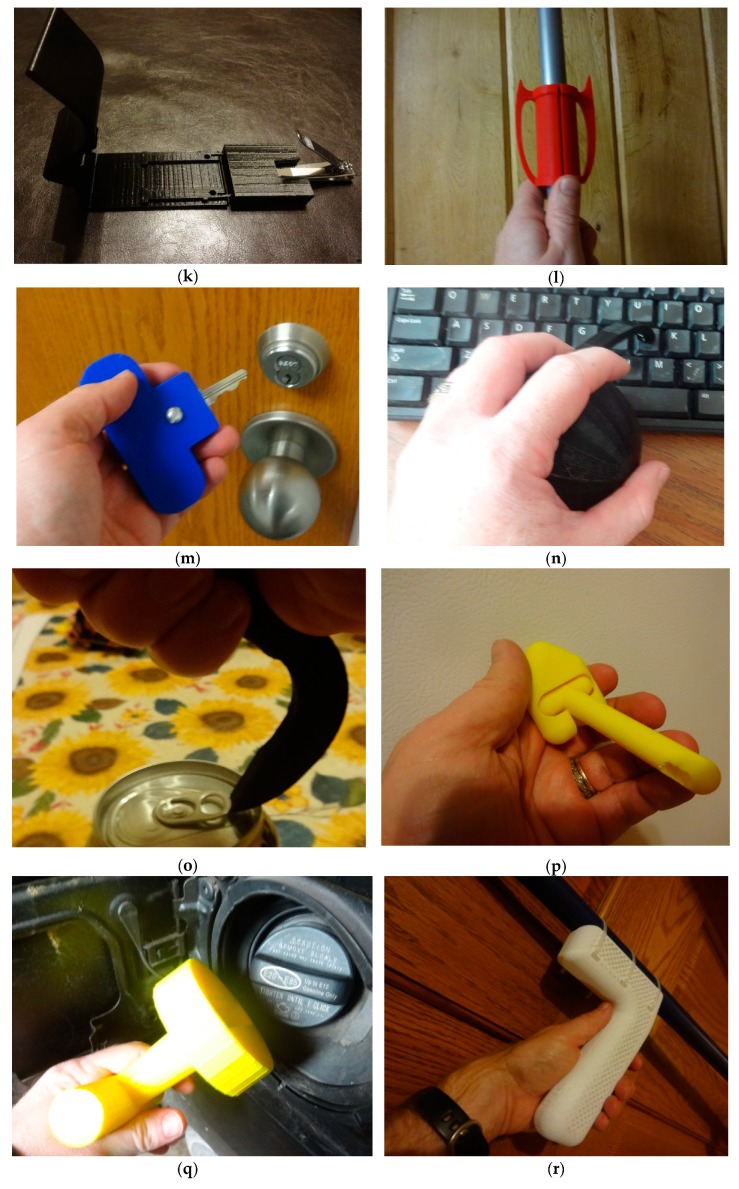

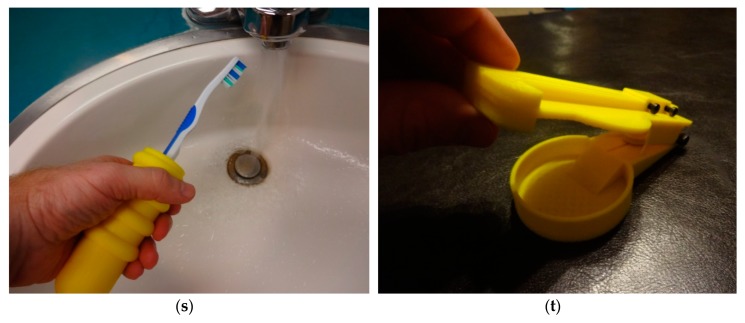
Twenty selected case-study arthritis adaptive aids from Table 1 (**a**) scissor aid; (**b**) car seat aid; (**c**) drawer aid; (**d**) light switch aid; (**e**) sock aid; (**f**) knife guide aid; (**g**) phone holder aid; (**h**) zipper pull aid; (**i**) knitting aid; (**j**) pen holder aid; (**k**) nail clipper aid; (**l**) cane holder aid; (**m**) key aid; (**n**) typing aid; (**o**) pop can aid; (**p**) dog leash aid; (**q**) gas cap aid; (**r**) handle adapter aid; (**s**) toothbrush aid; (**t**) pill splitter aid.

The twenty selected case-study arthritis adaptive aids from Table 1 were successfully 3-D printed on delta style RepRap 3-D printers and are shown in Figure 1.

All of the aids shown in Figure 1 were able to perform their primary functions as adaptive aids. This means that each device was able to perform the technical mechanical function for which it was designed, but further work is needed to ascertain over what patient populations (e.g., severity of arthritis) the devices would be applicable. The adaptive aids were printed in various colors to demonstrate one level of customization that would be available to makers with no additional cost (e.g., patients could get all their adaptive aids to match their home décor, favorite color or clothing).

The energy use of the 3-D printer was found to be about 40 W during printing. With energy use during translation (movement without printing of the head) and warming up to printing temperature included, the 3-D printer uses about 0.06 kW/h or less than 1 penny per hour to operate. As the filament costs dwarfed the electricity costs they were neglected for simplicity. Lastly labor costs were also excluded from the financial analysis because fabricating an aid with a 3-D printer would fall under self-consumption (e.g., be treated in the same way by an arthritis patient to cooking their own microwave dinner, where labor and/or opportunity costs are not considered). This assumption is valid for pre-designed arthritis aids (following Section 2.3 criteria 1) using the 3-D printer in this study (or equivalent). This is possible because the time it takes to search for the 3-D printable design of an arthritis aid is approximately equivalent to the time needed for shopping for the equivalent product on line. Any time needed for customization of the aid (e.g., to fit one’s hand size following Section 2.3 criteria 2) is also approximately the same as taking measurements and inputting them in to select the right size commercial product. The time it takes to order a commercial adaptive aid (i.e., input payment methods such as credit card information and shipping address) is about the same time it takes to download an STL, choose printer settings and start the necessary print jobs in distributed manufacturing (following Section 2.3 criteria 4). The actual print time is not a factor in the economics as the filament cost dominates the operating costs and the printers can be comfortably operated without observation or labor intervention. As noted in Section 2.2, there is no post processing of the 3-D printed components and the user simply has to remove them from the print bed and assemble. This step can be looked at as a time investment less than that needed for unpacking a shipped online order and assembling it. The assembly of the twenty products needed no special skills or equipment and could be accomplished by the average consumer. All of the 3-D printed components were massed after printing and the results are shown in Table 2.

As can be seen in Table 2, using distributed manufacturing to fabricate adaptive aids can save arthritis patients considerable financial resources. The financial savings for the patients ranged from 86.3–98.5% per product manufactured with a consumer desktop 3-D printer, with average savings of over 94%. Overall, the 20 example adaptive aids could be printed for a 1 kg of plastic, which costs $20, while on average each single adaptive aid would save the patient more than $20. 3-D printing all 20 adaptive aids would save about $400, which means only 30 adaptive aids alone could fully cover the capital cost of the printer along with the filament and electricity costs.

The mass of the adaptive aid is highly dependent on slicing settings used for 3-D printing. However, based on the results of the 150 replicates of an ASTM standard from thirty new 3-D printer users that resulted in a standard deviation of 1.58 and an envelope within +/−10% of the average, it can be concluded that the cost of the adaptive aid will vary at most by 10% from new user setting variations. This does alter the percent savings but does not affect the conclusions. For example, the toothbrush aid had a mass of 69.55 g. If it was printed with a mass of 76.5 g (a 10% increase) it would result in a cost of US$1.53, which is a 91.8% savings rather than 92.5%.

## 4. Discussion

### 4.1. Distributed Manufacturing Economics

Similar to previous work that has shown substantial economic savings for 3-D printing high-end scientific tools [77,78,79,80,81,82], it is clear that distributed manufacturing can radically reduce the cost of producing adaptive aids for those with arthritis. Despite the relatively high proportion of the population with arthritis (particularly the geriatric population), the market for adaptive aids is still much smaller than conventional consumer products and thus economics of scale have not driven down the costs of adaptive aids. This savings potential is also likely because similar to scientific equipment that often needs to be quasi-personalized, the ability of the 3-D printer to make a bespoke product for a specific problem for someone with arthritis provides the highest value. All of the designs shown in Figure 1 were either made with OpenSCAD (http://www.openscad.org/) or FreeCAD (https://www.freecadweb.org/), both of which are natively parametric CAD packages allowing relatively easy customization for those with access to the raw source files. This was the case for all the designs selected because of criteria 2. In addition, customizer applications have been developed for OpenSCAD that enabled novices to customize products with no knowledge or experience with CAD because of a simple user interface [83]. This makes the designs accessible even to those with only modest technical and computer competency. 3-D printing itself has also become considerably more user friendly and for the use of standard materials (like the PLA used in this study—criteria 5) and straightforward prints, the files are easily printed akin to printing a document on a conventional 2-D printer.

Ninety-four percent savings are substantial enough for a person with arthritis to consider gaining access to a 3-D printer, whether purchasing and operating it themselves, using one at the local library, makerspace, fablab, or an online service (e.g., Shapeways). Thus, it should be clear that although the highest level of savings are for patients to print their own aids, those with arthritis can still get 3-D printed aids from any number of 3-D printing services including major retailers and shippers as well as local 3-D printing on demand services such as MakeXYZ. It should be pointed out that the markup for commercial online services could erode the savings shown here, but would depend on the service as well as provide a wider selection of materials (e.g., metal) that may make for a more robust aid. It is clear that the savings per adaptive aid are high enough that margins could be maintained by also including labor costs although this would be highly variable depending on the community. All of the adaptive aids shown in Figure 1 can be fabricated reasonably easily with a desktop 3-D printer and basic assembly skills (e.g., no machinist or other specialty skills are necessary). However, it should be pointed out that someone suffering from severe hand arthritis may need assistance assembling (e.g., tightening down M3 nuts and bolts for some of the designs like the scissor aid (Figure 1a), knife holder (Figure 1f), key holder (Figure 1m), and pill splitter (Figure 1t)).

Although some of the designs were developed by those with arthritis themselves, many of the arthritis aids available on the Internet were made by makers that know patients or by groups like Makers Making Change. It should be pointed out that the 20 case study adaptive aids used here are a tiny fraction of the aids of all kinds already available in the numerous 3-D printing free design repositories that now hold millions of designs of all manner of products [34]. It should also be noted that for some of these adaptive aids, a patient would want more than one copy of the aid (e.g., drawer, light switch, key, and handle adapter aids) and thus the twenty example case study products shown here could provide the capital savings alone for the purchase of one of the lower-cost fused filament-based 3-D printers.

### 4.2. Limitations and Future Work

This was a preliminary investigation into the technical and economic potential for adaptive aids for arthritis patients that specifically focused on arthritis of the hands and thus could be significantly expanded in the future. First, designs for adaptive aids for other types of arthritis could be similarly evaluated. Second, an entire catalog of open-source 3-D printable equivalents for all commercial adaptive aids could be developed and then submitted to the same study as was done here. The economic analysis here assumed a similar lifetime for the 3-D printed products and the conventionally manufactured products. This assumption would need to be carefully analyzed and the products tested over long time periods to determine lifetimes to do full life cycle economic analysis. Many products like the light switch extender (Figure 1d), pen holder (Figure 1j) or toothbrush holder (Figure 1s), which do not undergo significant mechanical stresses would likely perform over long periods the same as an injection-molded component, however, others that undergo significant stresses may not be as robust (e.g., the pill splitter shown in Figure 1t). The pill splitter design, for example, may need to be optimized by both removing the bolts and using filament rivets, which would eliminate the cost of fasteners, while at the same time adding in a metal blade instead of a replaceable polymer one (that would increase the cost). Like all free and open-source projects the designs shown here, although certainly technically useable, could be further improved and the open-source nature of them is an invitation to do just that [84,85,86]. This concept of continual improvement and innovation is a challenge in healthcare [87]. The nature of adaptive aids being consumer products meant to improve the quality of life of patients may be the first step into applying the more rapid innovation cycles well-established in the open source technology design world to medical care.

The largest savings are generally seen for products that can be completely 3-D printed. Some of the examples shown here such as pen holder aid (Figure 1j), typing aid (Figure 1n), light switch aid (Figure 1d), and car seat aid (Figure 1b). The car seat aid for example makes use of a 3-D printed living hinge, which is an advanced technique for changing the rigidity of different parts of a 3-D printed component to allow a higher function. Future designs should make of such innovations to reduce the need for non-printed components in a design. Further work is also necessary to go beyond adaptive aids for hand arthritis and could for example look at those to improve mobility, which has been shown to be a major factor in patient quality of life [88]. Further work is needed to better optimize the function of all the designs, making them easier to print, assemble, and use. Although all the adaptive aids analyzed in this study were able to perform their mechanical functions, further study is needed to have the devices evaluated by patients in different age groups to ensure they are technically proficient at performing their functions. If there are discrepancies found, they should be re-designed and tested.

Lastly, all economic analyses completed in this study focused on the U.S. market. It is likely that similar economic savings could be found for other regions of the world including Central and South America, Europe, Oceania, Asia, and Africa, but further work is needed to demonstrate this with the same detailed analysis. Many regions in the world (e.g., Europe) would have higher costs for imported items because of VAT or tariffs. This complicates the analysis because not only does the cost of the commercial adaptive aid change, but so does the cost of 3-D printing filament. Also, in many areas of the world with a less robust 3-D printer market, low-cost 3-D printing filament may not be as easily accessible. This lack of access can be offset with the use of recycled locally-manufactured filament [45], which would also further reduce the costs of manufacturing arthritis aids.

### 4.3. Applications for Both Old and Young People With Arthritis

Arthritis does not only take effect in elderly people above the age of 65, but also on otherwise healthy members of the younger generations. For example, Caroline Wozniacki, a professional tennis player, was diagnosed with rheumatoid arthritis at age 28, just a few weeks before the U.S. Open this year. Wozniacki spoke about how she is working with her diagnosis to overcome her hardships and work toward a goal of creating a positive mindset for herself [89]. A study from 2001–2004 by the National Ambulatory and Medical Care Survey, found there were approximately 294,000 children ages 0–17 years that were affected by arthritis or some other rheumatic condition [90]. A large population of the children in the U.S. that have these conditions may not have the tools or resources to work with their diagnosis as easily as a life-experienced adult or professional athlete. By using these 3-D printed aids from an open source file, such as the toothbrush holder (Figure 1s) or the zipper pull (Figure 1h), the children will not have as difficult of a time going about their daily routines.

The largest potential savings for the arthritis patients are if they are able to manufacture the aids themselves. Although arthritis can impair the young, arthritis relevance increases with age and the geriatric population is the one that would benefit the most from this technological development. However, it remains unclear if older patients would have the appropriate technological knowledge to 3-D print their own aids. Future work is necessary to evaluate this along with the ability of all ages of arthritis patients to search for and identify open source 3-D-printable designs online. A study could be done to confirm that the time it takes arthritis patients from all age groups to search for the 3-D-printable design online is approximately equivalent to the time needed for shopping for the equivalent product online as it is for experienced 3-D printer users. This future clinical trial could consider a significant number of patients, grouped in different grades of arthritis conditions who are asked to determine the difficulty to print out the aids, degree of satisfaction, useful of the aid, support received, self-capability to find objects in a catalogue or online, run the 3-D printer and fabricate the object, as well as their subjective cost to effort value received.

These readily 3-D printable objects can have a great effect on the community. For example, consider a person was leaving her apartment because her family came to pick her up. When she returns, she must figure out the best way of holding a small key and inserting it into the lock in the door. What if she had a Key Case (Figure 1m) that allowed her to both find her key faster in her purse because of its size and assisted her in turning the key to unlock the door. Not only are these designs easily accessible, but they are easily modified to accommodate the person for which the design is being made. This open source distributed manufacturing methodology has the potential to significantly alter the way the majority of arthritis sufferers adapt to their condition by providing a far more affordable option for many patients all over the United States (and potentially the rest of the world).

### 4.4. Deployment of 3-D Printers as Manufacturing Tools in Context

Arthritic diseases cause significant morbidity, costing patients between $5,700 and $9,600 yearly [91]. With an estimated 54.4 million adults in the USA suffering from arthritic conditions [92], the economic burden of medical costs for arthritic conditions was estimated at approximately $140 billion in 2013 [93] and is expected to grow as the population ages and as obesity affects a greater proportion of the population [92]. Patients suffering from arthritis often have difficulty accomplishing both occupational tasks as well as ADLs. Private health insurance covers some doctor-prescribed assistive aids and Medicare Part B covers certain aids as Durable Medical Equipment (DME). Even with such cost-sharing, Medicare patients are responsible for all costs until their deductible is reached and for 20% of costs as copayments thereafter. Assistive aids are thus a significant expense for patients. As the population of arthritis patients increases as discussed above, so too will the costs associated with assistive aids. As the results of this study show, all of the open-source adaptive aids can be manufactured for less than the 20% copayment and thus it can be inferred that 3-D printing adaptive aids will be economic for Medicare patients as well as those with no insurance.

The type of assistive aids required by an individual patient depends on which of the patient’s joints are affected by the arthritis, the severity of the patient’s arthritis, the frequency with which they need to accomplish a particular task, and the force needed to go through the patient’s joints to accomplish the task. Modifications for various tasks can be done by using an alternative tool (i.e., using an electric can opener as opposed to a manual one), by retrofitting a patient’s home with accessible features (i.e., elevated toilet seats, grab bars in the bathroom, and 3-D printable aids such as door handles as opposed to door knobs [94] and automotive transfer handles [95]), or by changing a tool to make it more comfortable or appropriate to the task (adding a handle to a key to allow a handgrip to turn the key as opposed to a key pinch grip by the finger and thumb as shown in Figure 1m). By using these strategies to modify activities that are painful for patients with arthritis, patients can perform tasks that they were previously not able to perform comfortably. This may lengthen the careers of arthritis patients and may also allow them to live independently at home for a longer period before requiring assisted living situations.

Deploying 3-D printers in doctor’s offices, where musculoskeletal complaints account for 13% of all visits, would allow for physicians to suggest, prescribe, and print properly customized and sized aids for their patients at the point of care when the patient presents with a complaint. This is one method by which patients could be educated on their disease and have their needs addressed in a practical manner by their physician without the need for opioids or other pharmaceuticals. Future work is needed to determine if this would create an increase in visits as patients want to customize, replace or obtain additional copies of a 3-D printed aid. In addition, how to provide compensation for the staff time needed to provide this service would need to be determined. In a similar way, 3-D printers could be very effectively used in physical therapists’ and occupational therapists’ clinics as many patients presenting to these health care providers have specific functional requirements for which they are seeking solutions. While strengthening the patient’s muscles and improving their joints’ range of motion will provide benefit to the patient, so too will the use of specific functional aids which the therapist could prescribe and customize to the patient in clinic before printing and fitting the aid to the patient.

Nursing homes and assisted living residences could also take advantage of having a 3-D printer on-site to be able to provide functional aids for their patients with physical limitations. Arthritis is a significant and underreported problem in nursing homes [96]. Patients with arthritis require more assistance with walking and ADLs than patients without a diagnosis of arthritis [96]. The nurses and nursing aides often observe patients having trouble with ADLs and struggle to provide assistance to all of the patients under their care. By providing patients with functional aids where they struggle with certain ADLs (for example, being unable to cut their food due to not being able to hold a knife properly but being able to cut their food with a knife modified with a large handle to enable a grasp hold on the knife or using a knife guide aid as shown in Figure 1f), the patients may retain aspects of independence longer, have improved self-esteem, and free the staff from certain helping tasks.

Hospitals would also be able to offer their patients the benefit of 3-D printed assistive devices. Many patients present to hospital with new acute conditions, but also with undiagnosed chronic conditions as many of the elderly who live alone have either found a method of coping with their disabilities or simply avoid certain activities that they cannot accomplish independently. Upon evaluation in hospital, the opportunity arises to address some of these deficiencies and improve the patient’s level of function and activity. Arthritis is the most expensive condition treated in hospital by private health insurance in the U.S., and the second most expensive condition treated in U.S. hospitals overall in 2013 [97]. Hospitals could provide assistive devices to deal with temporary acute disabilities (such as from fractures or from joint replacement surgeries) as well as devices to help with chronic dysfunctions.

There is a gap found in surveys between actual assistive device usage and perception of personal benefit from assistive devices for ADLs in patients with disability [98]. The reason for this gap has not been fully elucidated. This gap could exist because of mental challenges, such as depression or social embarrassment from having arthritis. If embarrassment is playing a core role preventing people from purchasing adaptive aids, being able to manufacture them in their own homes may overcome this barrier to adoption. It is also possible that financial considerations are primarily responsible for patients not obtaining all the assistive devices that they believe would be effective in improving their function. If this is the case, lowering the cost of obtaining assistive devices by distributed manufacturing of free and open source designs would have a significant impact on the lives of patients disabled by arthritis and would have a significant beneficial effect on society. Other reasons for patients not to obtain all prescribed assistive devices might be that the off-the-shelf devices may only partially improve the task for the patient as the device does not allow for modifications based on the physical characteristics of the patient, such as hand size. In this case, the open source customizable designs offer the potential to make the devices perfectly sized to an individual patient. Thus, 3-D printing of customizable free and open source assistive devices is able to improve access and function to assistive devices to disabled patients who may be more likely to benefit then from these devices.

## 5. Conclusions

As the results of twenty freely accessible case-study designs show, adaptive aids for those that suffer from arthritis can be successfully fabricated on low-cost desktop 3-D printers and are technically proficient at performing their functions. On average, distributed manufacturing of these open-source adaptive devices resulted in financial savings greater than 94% compared to commercially-available products. Thus Medicare patients are able to 3-D print adaptive aids for less than their 20% copayments on prescribed DMEs. Remarkably, a single spool of polymer filament that retails for less than US$20 could print out all the aids investigated in this study and, on average, each adaptive aid would save the user over US$20. As printing a tiny subset of the available adaptive aid designs freely available on the internet or those needed by even a single patient would recover the full capital and operational costs of a low-cost 3-D printer, it can be concluded that there is considerable potential for distributed manufacturing to assist arthritis patients. This is perhaps most clear in nursing homes and hospitals where the capital costs for 3-D printers as well as the expertise to use them could be amortized over a large population of patients. As shown in this study, open access designs for arthritis aids are readily available and accessible. These designs help with a variety of tasks. With the advancements of open-source devices like those shown here, makers can help to alleviate some of the burden and pain that arthritis symptoms may cause. Policy makers as well as philanthropists should consider funding the open-source design of arthritis aids that have not already been developed. As arthritis is going to be an even more prevalent problem in the future, the application of distributed manufacturing represents a means to economically help society adapt to some of the increased costs associated with the disease.

## Figures and Tables

**Table 1:** Selected open-source 3-D printable adaptive aids for arthritis patients.

Adaptive Aid	Description	CAD	Source
Scissor aid	Enables the use of scissors using palm for cutting and fist for opening.	FreeCAD	[57]
Car seat aid	Car seat buckle depressor using a living hinge.	FreeCAD	[58]
Drawer aid	Drawer and cabinet opener with built in can opener.	FreeCAD	[59]
Light switch aid	Easier access for turning on and off light switches.	FreeCAD	[60]
Sock aid	An assistive aid for putting on socks that will not slip on carpet.	OpenSCAD	[61]
Knife guide aid	Used to cut up ingredients with large handle attached.	FreeCAD	[62]
Phone holder aid	Attaches to write for the easy accessibility of your phone.	FreeCAD	[63]
Zipper pull aid	Used to pull up zippers of necessary clothing items.	FreeCAD	[64]
Knitting aid	Large blocks for a better grip on the knitting needles.	OpenSCAD	[65]
Pen holder aid	Provides a larger area that encompasses the pen to hold in hand.	OpenSCAD	[66]
Nail clipper aid	Provides easy access to clip one’s nails by using the palm of your hand.	OpenSCAD	[67]
Cane holder aid	Allows the user to hang their cane for easy access.	FreeCAD	[68]
Key aid	Attaches to the users key for a larger surface area to grab on to.	FreeCAD	[69]
Typing aid	Round ball can be customized for palm size and allows user to type on keyboard easier.	OpenSCAD	[70]
Pop can aid	Assists in the opening of pop cans by use of a hook.	OpenSCAD	[71]
Dog leash aid	Uses a “lock and key” method to hold onto your dog’s leash.	OpenSCAD	[72]
Gas cap aid	Easy grip handle to be able to open gas cap on car.	OpenSCAD	[73]
Handle adapter aid	Ability to attach to any handle for easier use and grip.	FreeCAD	[74]
Toothbrush aid	Enables user to grab their toothbrush more easily. Can be customized for hand size.	OpenSCAD	[75]
Pill splitter aid	Users can use their palm to push down and split their medication.	FreeCAD	[76]

**Table 2:** Economic comparison of distributed manufacturing and commercial arthritis adaptive aids.

Adaptive Aid	Mass (g)	Distributed Manufacturing Cost (US$)	Purchase Cost (US$)	Savings (US$)	Savings (%)
Scissor aid	116.19	2.62	31.95	29.33	91.8
Car seat aid	16.36	0.33	14.99	14.66	97.8
Can opener aid	27.50	0.55	9.99	9.44	94.5
Light switch aid	19.98	0.40	17.99	17.59	97.8
Sock aid	161.16	3.22	43.95	40.73	92.7
Knife guide aid	37.61	0.95	12.95	12.00	92.7
Phone holder aid	36.77	0.79	49.99	49.20	98.4
Zipper pull aid	22.62	0.45	19.75	19.30	97.7
Knitting aid	88.07	1.96	16.99	15.03	88.4
Pen holder aid	11.14	0.22	9.95	9.73	97.8
Nail clipper aid	50.75	1.01	10.95	9.94	90.8
Cane holder aid	25.00	0.50	14.95	14.45	96.7
Key aid	20.66	0.41	9.10	8.69	95.5
Typing aid	70.25	1.40	49.95	48.55	97.2
Pop can aid	20.03	0.45	5.99	5.54	92.5
Dog leash aid	14.92	0.30	19.99	19.69	98.5
Gas cap aid	102.42	2.05	14.95	12.90	86.3
Handle adapter aid	85.89	1.72	21.95	20.23	92.1
Toothbrush aid	69.55	1.40	18.79	17.39	92.5
Pill splitter aid	53.55	1.27	23.75	22.48	94.7

## Supplementary Materials

The following are available online http://www.mdpi.com/2308-3417/3/4/89/s1, Table S1: Spreadsheet of equivalent commercial adaptive aids.
